# 2-Triazenoazaindoles: A novel class of triazenes inducing transcriptional down-regulation of *EGFR* and *HER-2* in human pancreatic cancer cells

**DOI:** 10.3892/ijo.2011.1272

**Published:** 2011-11-29

**Authors:** JAN N. KREUTZER, ALESSIA SALVADOR, PATRIZIA DIANA, GIROLAMO CIRRINCIONE, DANIELA VEDALDI, DAVID W. LITCHFIELD, OLAF-GEORG ISSINGER, BARBARA GUERRA

**Affiliations:** 1 Department of Biochemistry and Molecular Biology, University of Southern Denmark, Odense, Denmark; 2 Department of Pharmaceutical Science, University of Padova, Padova; 3 Department of Molecular and Biomolecular Science and Technologies, University of Palermo, Palermo, Italy; 4 Department of Biochemistry and Oncology, Schulich School of Medicine and Dentistry, University of Western Ontario, London, Ontario, Canada

**Keywords:** 2-triazenoazaindoles, pancreatic cancer, cell death, EGFR, HER-2

## Abstract

Pancreatic cancer is a complex malignancy arising from the accumulation of genetic and epigenetic defects in the affected cells. Standard chemotherapy for patients with advanced disease shows only modest effects and is associated with considerable toxicity. Overexpression or aberrant activation of members of the epidermal growth factor receptor tyrosine kinase family, which includes EGFR and HER-2, occurs frequently and is associated with multiple drug resistance and decreased patient survival. In this study, we have investigated the therapeutic potential of AS104, a novel compound of the triazene class, with potential inhibitory effects on EGFR. We found that treatment of cells with AS104 causes significant reduction of cell growth and metabolic activity in four human pancreatic cancer cell lines. Furthermore, we show that the AS104-mediated induction of apoptotic cell death is associated with stimulation of autophagy in a dose-dependent manner. Treatment of cells with AS104 results in significant down-regulation of EGFR and HER-2 expression and activity and subsequent inhibition of downstream signaling proteins. Quantitative RT-PCR analysis and assays with proteasome inhibitors revealed that AS104 regulates the expression of EGFR and HER-2 at the transcriptional level. These findings provide for the first time experimental evidence for efficacy of AS104 in the simultaneous transcriptional repression of *EGFR* and *HER-2* genes and suggest that AS104 may have therapeutic potential in the treatment of pancreatic cancers that express high levels of the aforementioned receptor tyrosine kinases.

## Introduction

The majority of malignant tumors affecting the exocrine pancreas are histologically defined as pancreatic ductal adenocarcinomas. These types of tumor rapidly grow and metastasize representing one of the leading causes of cancer-related death in developed countries (1,2). Current therapeutic treatments for patients with advanced disease that are not suitable for surgical resection have only modest effects due to low response rates, substantial toxicity and a median survival of less than six months (3,4). In this respect, numerous attempts have been made with several chemotherapeutic drugs, however, with little success because pancreatic cancer shows an inexplicable drug resistance towards platinum agents, taxanes and topoisomerase inhibitors (5). The molecular mechanisms associated with drug resistance in pancreatic cancer are subject of intense investigations although loss of p53 function, deregulated Ras signaling and altered phosphatidylinositol-3-kinase (PI3K)/AKT pathway may play a major role (6,7).

Currently, it is generally accepted that one of the major factors contributing to the resistance of pancreatic cancer cells to treatment with chemotherapeutic agents is represented by the autocrine epidermal growth factor (EGF)-mediated signaling which results in stimulation of the PI3K pathway and is required for transformation of several cell lineages by *RAS*-family oncogenes (8,9). Consistent with the existence of the aforementioned autocrine loop is the notion that pancreatic cancer cells overexpress EGF-family ligands and receptors (EGFR, HER-2 and -3) (10,11). The EGFR protein also known as ErbB1, belongs to a family of transmembrane tyrosine kinase growth factor receptors whose stimulation by ligand binding results in the activation of the MAPK-, PI3K/AKT pathways, transcription factors and signal transducers (1). EGFR is expressed in 30–89% of pancreatic cancers and its aberrant expression has been shown to correlate, in some cases, with worse outcome and more aggressive disease. Interestingly, in xenograft models of pancreatic cancer, the combination of gemcitabine and EGFR-targeted therapy significantly inhibited the metastatic process and resulted in improved overall survival (12,13). A major partner of EGFR is HER-2 (ErbB2) whose activation is dependent on dimerization with other members of the aforementioned family of receptor tyrosine kinases. Aberrant expression of HER-2 in pancreatic cancer has been reported in a number of studies and associated with resistance to various chemotherapeutic agents (14,15). Recently, improved understanding of the molecular mechanisms responsible for the acquired or inherent resistance of pancreatic cancer cells towards EGFR- or HER-2 targeted therapy suggested that combination therapy based on agents targeting both receptors and/or multiple pathways supporting proliferation of cancer cells might represent a more efficacious treatment approach towards this disease.

**Figure 1:**
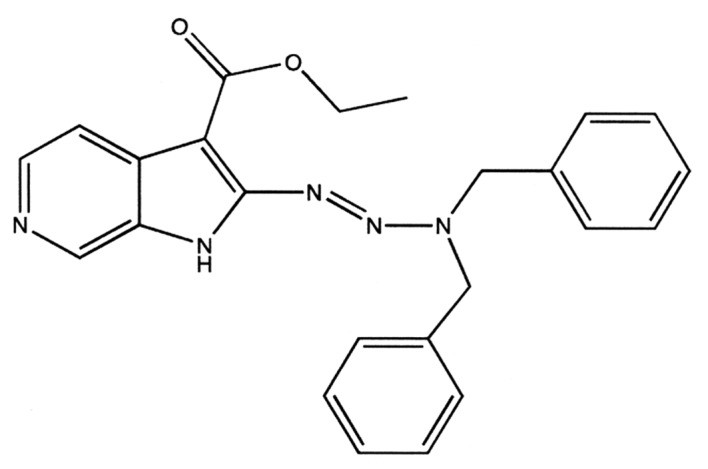
The structural formula of AS104.

A preliminary screening with a panel of compounds bearing a 2-triazenoazaindole scaffold (16), expected to inhibit EGFR kinase activity and exert cytotoxic effects towards cells carrying aberrant expression of EGFR, led to the identification of a novel low molecular-weight agent, i.e., ethyl 2-(3,3-dibenzyl 1-triazenyl)-1H-pyrido(2,3-c)pyrrolo-3-carboxylate (AS104), showing significant anti-proliferative effects (Fig. 1). In the present study, in view of these preliminary findings we examined the biological and biochemical effects of AS104 on pancreatic cancer cells notoriously resistant to gemcitabine treatment (17,18) and showing aberrant expression of EGFR and HER-2 tyrosine kinases, respectively. We report for the first time evidence that simultaneous targeting of these receptors may represent an effective strategy for overcoming treatment resistance of pancreatic cancer cells.

## Materials and methods

### Cell culture and treatments

The pancreatic ductal adenocarcinoma PANC-1, BxPC-3, Capan-1 and MiaPaCa-2 cell lines and the human osteosarcoma U2OS cell line were purchased from the American Type Culture Collection (ATCC), cultured according to the growth conditions recommended by the supplier and maintained at 37°C in a humidified atmosphere containing 5% CO_2_. Cells were treated with DMSO (the final concentration was 0.2% in all experiments), doxorubicin (Sigma) or AS104 (16) as described in the figures legend. Where indicated, cells were incubated with 100 ng/ml human recombinant epidermal growth factor (Sigma) for 10 min after 24 h starvation in serum-free medium or 50 μM MG132 (Calbiochem) for 6 h.

### Determination of cell viability, proliferation and clonogenic survival

The WST-1 viability assay (Roche) was performed in 96-well plates. Twenty-four hours after seeding, cells were treated with DMSO or various concentrations of AS104 for 48 h. WST-1 reagent was added to the cells and cell viability was determined as previously described (18). Cell proliferation was determined by the Cell Proliferation Assay (Calbiochem) following the manufacturer’s instructions and as reported earlier (18). For the clonogenic survival assay, cells were treated with various concentrations of AS104 for 24 h. Afterwards, cells were trypsinized and seeded in 6-well plates and colonies were allowed to grow for 14 days. Subsequently, cells were washed in PBS and stained with a solution containing 0.1% crystal violet in 20% ethanol and 0.1 M sodium borate and destained with dd water. Staining of the colonies was then quantified by subsequent re-solubilization of crystal violet dye in the presence of methanol and reading of the difference in absorbance at 540 nm wavelength.

### Flow cytometry

Cells treated according to the conditions indicated in the figure legends were collected by trypsinization, washed with PBS and fixed for 24 h with 70% ethanol at -20°C. For the determination of cell death (i.e., the sub-G1 region which comprises cells with reduced DNA in late apoptosis or necrosis), cells were incubated with 20 μg/ml propidium iodide (Sigma) and 40 μg/ml RNAse A (Sigma) in PBS for 30 min at room temperature. To detect the formation of acidic autophagic vacuoles, control or treated cells were stained with 1 μg/ml acridine orange (Sigma) for 15 min at 37°C, removed from the plates by trypsinization and immediately afterwards analyzed by flow cytometry. Cells were analyzed on a FACSCalibur flow cytometer (Becton-Dickinson) and data were processed using the CellQuest™ Pro software. For each measurement, 10,000 events were analyzed.

### Preparation of cell extract and Western blot analysis

Cell lysates were prepared as previously described (19). Proteins were detected by probing Western blot membranes with the following antibodies: mouse monoclonal anti-poly (ADPribose)polymerase (PARP), anti-mTOR, anti-AKT (all from BD Biosciences); rabbit polyclonal anti-p44/42MAPK, rabbit monoclonal anti-phospho-p44/42MAPK (T202/Y204), rabbit polyclonal anti-phospho-AKT (T308), mouse monoclonal anti-phospho-AKT (S473), rabbit polyclonal anti-LC3B, rabbit polyclonal anti-HER-2, rabbit polyclonal anti-phospho-HER-2 (Y1221/1222), rabbit polyclonal anti-cytochrome c (all from Cell Signaling Technology); mouse monoclonal anti-Myc, rabbit polyclonal anti-EGFR, goat polyclonal anti-phospho-EGFR (Y1173), mouse monoclonal anti-ATP5B (all from Santa Cruz Biotechnology); mouse monoclonal anti-β-actin (Sigma). Protein-antibody complexes were visualized by chemiluminescence using CDP-Star (Applied Biosystems) as a substrate according to the manufacturer’s recommendations.

### Isolation of mitochondria from whole cell lysate

Cells were harvested by trypsinization, washed with ice-cold PBS, counted and adjusted to 10^6^ cells/sample. Subsequently, they were re-suspended in lysis buffer (19). Isolation of mitochondria was carried out by the Mitochondria Magnetic Isolation kit (Miltenyi Biotech) following the manufacturer’s instructions.

### Quantitative real-time PCR

Total RNA from cells treated as indicated in the figure legends was isolated using the RiboPure™ kit (Ambion). Total RNA (1 μg) was treated with DNAse I kit (Invitrogen) and used for reverse transcription using the Cloned AMV FS Synthesis kit (Invitrogen). cDNAs were then used as a template for the subsequent PCR. PCR reactions were performed in a 20-μl total volume consisting of 30 ng template, 1X SYBR^®^ Green JumpStart™ Taq ReadyMix™ (Sigma), forward and reverse primers relative to the cDNA of the analyzed proteins (200 nM forward and reverse primers for HER-2 and β-actin; 300 nM forward and 150 nM reverse primers for EGFR). All samples were prepared in triplicate. The reactions consisted of a 10-min initial denaturation (95°C) followed by 40 cycles of denaturation (95°C, 15 sec) and annealing/extension (60°C, 1 min for HER-2 and β-actin; 65°C, 1 min for EGFR). Measurement of EGFR-, HER-2- and β-actin gene expression levels was carried out according to the quantification method of the StepOnePlus Real-Time PCR System (Applied Biosytems). All mRNA expression values are ratios relative to β-actin. Fold changes in samples derived from drug-treated cells were determined relative to DMSO-treated samples. Primer pairs were as follows: for EGFR, forward primer 5′-GGCACTTTTGAAGATCATTTTCTC-3′ and reverse primer 5′-CTGTGTTGAGGGCAATGAG-3′; for HER-2, forward primer 5′-CCAGGACCTGCTGAACTGGT-3′ and reverse primer 5′-TGTACGAGCCGCACATCC-3′; for β-actin, forward primer 5′-GACAGGATGCAGAAGGAGATTACT-3′ and reverse primer 5′-TGATCCACATCTGCTGGAAGGT-3′ (20,21).

### Protein kinase profiling

The activity of protein kinases shown in Table I was tested against 10 μM AS104 by Reaction Biology Corp. (RBC) using a radioactive-based assay as described by the manufacturer. The ATP concentration employed by RBC was 10 μM. Residual kinase activities are expressed as the percentage of control activity measured in the absence of compound.

### Statistical and densitometric analysis

The statistical significance of differences between the mean of two sets of data was evaluated by the two-tailed t-test (Student’s t-test). The levels of significance are indicated in the figures legend. Quantification of the intensity of protein bands on Western blots was carried out with the ImageJ software (NIH).

## Results

### Analysis of AS104 effects on viability, proliferation and survival of human pancreatic cancer cells

**Figure 2:**
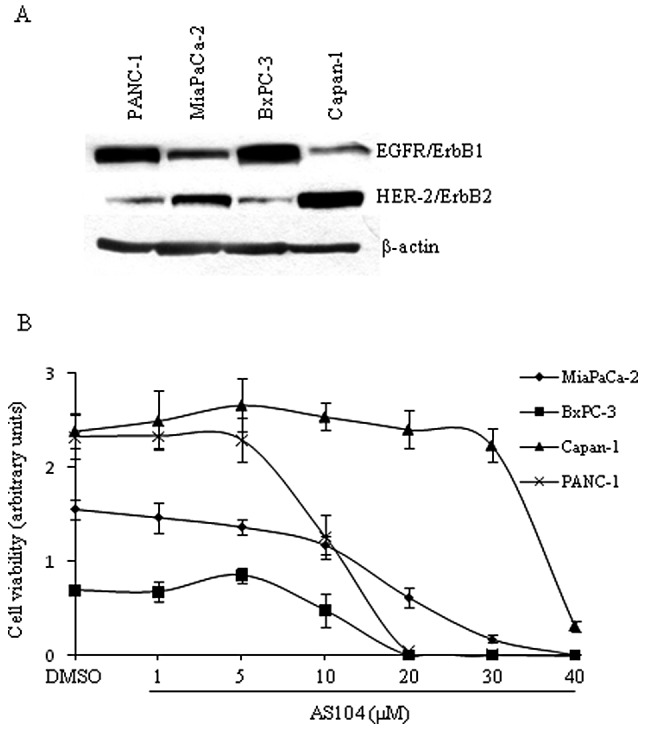
Effect of AS104 on viability of four human pancreatic cancer cell lines. (A) Protein expression levels of EGFR and HER-2 were analyzed by Western blotting on total cell lysates derived from the indicated cell lines. β-actin was used as loading control. (B) Cells were treated with DMSO and various concentrations of AS104 for 48 h, respectively. Proportion of viable cells measured by the WST-1 assay is indicated in arbitrary units as a difference in absorbance determined at 450 and 690 nm (reference) wavelengths, respectively. Three independent experiments were performed and data from one representative experiment [mean ± standard deviation (SD) of four replicates] are shown.

**Figure 3:**
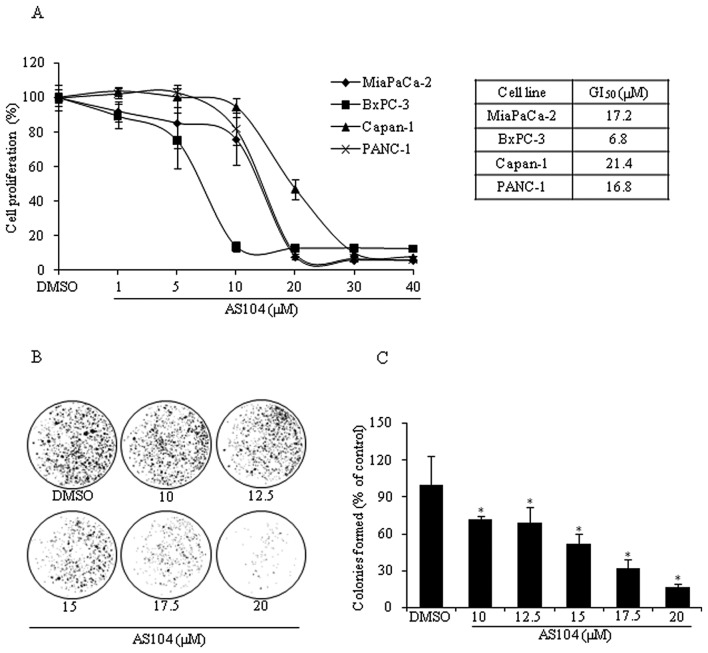
AS104 impairs proliferation and survival of human pancreatic cancer cells. (A) Cell proliferation of four human pancreatic cancer cell lines was determined by BrdU incorporation into genomic DNA. Results are expressed as percentage relative to the corresponding controls represented by cells incubated with DMSO. Insert to the right, shows the concentrations of AS104 inducing 50% growth inhibition (i.e., GI_50_) of the various cell lines after 48 h incubation with the drug. Three independent experiments were performed obtaining similar results and data from one representative experiment (mean ± SD of four replicates) are shown. (B) PANC-1 cells were incubated with DMSO and increasing concentrations of AS104, respectively, for 24 h. Cells were allowed to form colonies for 14 days. Colonies were visualized by staining with crystal violet as described in Materials and methods. Representative pictures of PANC-1 cell colonies after staining are shown. (C) Bar graph shows quantification of cell colonies. Average values ± SD from six independent experiments are shown relative to DMSO-treated control. ^*^Statistical significant difference in number of colonies formed after treatment with increasing concentration of AS104 as compared to the treatment with DMSO alone (Student’s t-test, P<0.05).

In our initial study, we examined the expression levels of EGFR as well as HER-2 on whole cell lysates from four human pancreatic cancer cell lines under basal conditions (Fig. 2A). We found that PANC-1 and BxPC-3 cell lines expressed significant higher levels of EGFR than MiaPaCa-2 and Capan-1 cell lines which, in contrast, displayed the highest expression levels of HER-2 in agreement with data reported earlier (22,23). Next, the four human pancreatic cell lines were employed for testing the cytotoxicity of AS104. The amount of metabolically active cells treated in the absence and presence of AS104, respectively, was determined as shown in Fig. 2B. Significant cytotoxicity was observed at concentrations ≥10 μM for three cell lines. Capan-1 cells showed a significant reduction in viability at 40 μM AS104. Moreover, analysis of the dose-response did not reveal a correlation between degree of cytotoxicity and level of expression of either EGFR or HER-2. The anti-proliferative effects of AS104 were investigated by measuring BrdU incorporation into newly synthesized DNA of actively replicating cells (Fig. 3A). AS104 inhibited the proliferation of all four cell lines in a dose-dependent manner irrespective of the levels of EGFR and HER-2. Growth inhibition (50%) (i.e., GI_50_) was achieved at concentrations ranging from 6.8 μM as in the case of BxPC-3 cells to 21.4 μM as for Capan-1 cells 48 h after treatment. As the analysis of viability and proliferation showed that all four cell lines were sensitive to the effects of AS104, subsequent experiments were carried out with one representative cell line: i.e., PANC-1. The survival ability of PANC-1 cells was assessed by performing a clonogenic survival assay (Fig. 3B and C). Cells incubated for 24 h with increasing concentrations of AS104 agent were allowed to form colonies for up to 14 days, which were revealed, afterwards, by crystal violet staining. Results indicated a survival rate of about 50% for cells incubated with 15 μM AS104 and the percentage of formed colonies rapidly decreased to ~16% when cells were treated with 20 μM AS104.

### Treatment of cells with AS104 leads to simultaneous induction of apoptosis and autophagy

**Figure 4:**
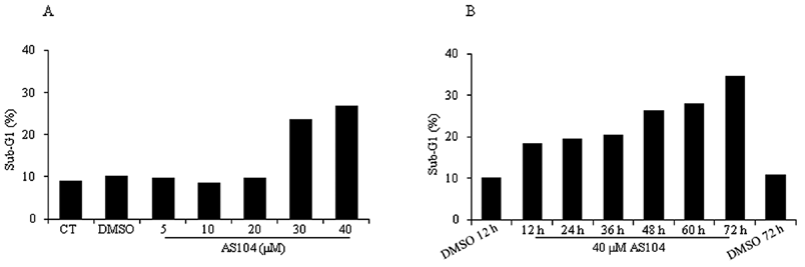
Incubation of PANC-1 cells with AS104 induces significant cell death. (A) Cells were left untreated (CT), incubated with DMSO or with various concentrations of AS104 for 48 h. Cells fixed and subsequently stained with propidium iodide were analyzed by flow cytometry. The fraction of dead cells (sub-G1) is expressed in percentage. Three independent experiments were performed obtaining similar results and data from one representative experiment are shown. (B) Representative time-course experiment performed by treating cells with either DMSO or 40 μM AS104 for the indicated time. Cells were analyzed by flow cytometry as described above. Experiments were performed three times obtaining similar results.

**Figure 5:**
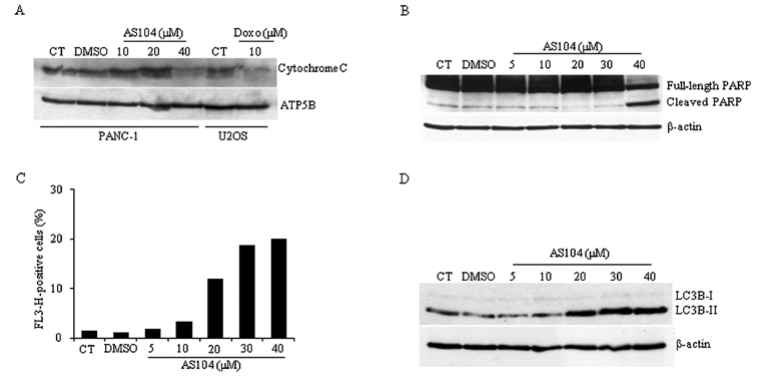
Treatment of PANC-1 cells with AS104 leads to combined induction of apoptosis and autophagy. (A) PANC-1 cells treated with the indicated concentrations of AS104 for 48 h were subjected to mitochondria isolation. U2OS cells were left untreated or incubated with doxorubicin for 24 h before harvesting and mitochondria extraction. Western blot analysis was performed using the indicated antibodies. The detection of ATP5B was carried out as a control for equal loading. (B) Cells were treated with AS104 as indicated in the figure for 48 h. Western blot analysis was performed for the detection of full-length (116 kDa) and cleaved (85 kDa) poly(ADPribose)polymerase (PARP). (C) Fluorescence-activated cell sorting analysis using the acridine orange-based assay was performed employing PANC-1 cells treated for 48 h as indicated in the bar graph. FL3-H indicates red-positive cells expressed in percentage. (D) Whole cell lysates from cells treated as indicated were subjected to Western blot analysis with antibodies directed against LC3B-I/II. All experiments were performed at least three times obtaining similar results. Data from one representative experiment are shown.

Next, flow cytometry analysis was conducted to measure induction of cell death in response to treatment with either increasing concentrations of AS104 for 48 h (Fig. 4A) or 40 μM AS104 and increasing incubation times (Fig. 4B). Incubation of cells with AS104 at concentrations ≥30 μM led to a significant accumulation of cells in sub-G1 (i.e., >20%) indicative of late-stage apoptosis or necrosis. Similarly, a time course experiment revealed significant cell death after 12 h of incubation with the drug (i.e., ~20%) and the percentage of dead cells increased to up to ~35% after 72 h. Treatment of cells with DMSO by itself led to a percentage of cell death comparable to the one deriving from untreated control cells (CT). To study whether cell treatment with AS104 would lead to an apoptotic type of cell death, cells were analyzed for the release of mitochondrial cytochrome c (Fig. 5A). A control experiment was carried out with the human osteosarcoma U2OS cell line incubated with 10 μM doxorubicin (Doxo) for 24 h as this compound has been shown to induce caspase-mediated apoptosis in this cell line (24,25). Detection of cytochrome c content from isolated mitochondria revealed that treatment with up to 40 μM AS104 for 48 h resulted in its decreased detection suggesting release of cytochrome c into the cytosol and hence caspase-mediated activation of apoptosis. Similar experiments were carried out investigating the caspase-3/caspase-7-mediated cleavage of PARP protein. The highest concentration of AS104 (40 μM) resulted in PARP cleavage in PANC-1 cells (Fig. 5B) supporting the notion that AS014 stimulates cell death through caspase activation, an early event during induction of apoptosis. Experiments conducted with the irreversible pan-caspase inhibitor z-VAD-fmk partially failed to counteract AS104-mediated cell death suggesting potential involvement of different types of cell death (data not shown). Therefore, we investigated whether treatment of cells with AS104 would stimulate autophagy. To detect and quantify the formation of autophagic vacuoles, cells were stained with acridine orange and subjected to flow cytometry analysis (Fig. 5C). AS104 increased red fluorescence up to 40 μM concentration with respect to control experiments, where an increase of up to ~20% positive signal was detected. Data obtained by flow cytometry were supplemented with results obtained by Western blot analysis of whole cell lysates (Fig. 5D). Conversion of LC3B-I into LC3B-II confirmed the induction of this catabolic process.

### Cell treatment with AS104 results in transcriptional down-regulation of the EGFR and HER-2 genes

**Figure 6:**
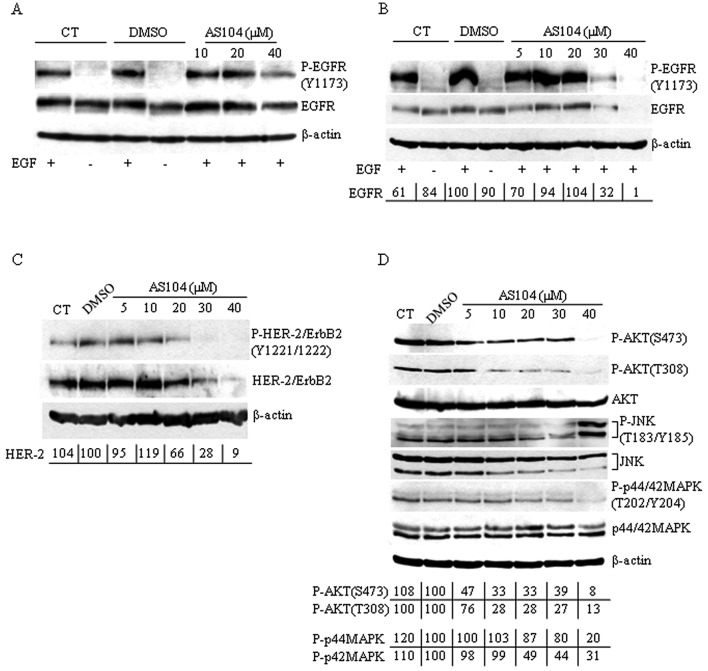
Cell incubation with AS104 leads to down-regulation of EGFR and HER-2 protein expression levels. (A) Total cell lysates (80 μg) from cells starved for 24 h in serum-free medium were left untreated, treated with DMSO or increasing concentrations of AS104 for 3 h. Where indicated, cells were stimulated with 100 ng/ml EGF for 10 min prior harvesting. Whole cell lysates were subjected to Western blot analysis with antibodies directed against EGFR or its phosphorylated form. (B) Experiments were performed essentially as described above except that cells were incubated with the drug for 48 h prior incubation with EGF. Whole cell lysates were analyzed by Western blotting with antibodies against the indicated proteins. Densitometric analysis of EGFR protein was performed with ImageJ software. Values below each band were normalized to control values from cells incubated with DMSO and EGF. (C) Experiments were performed as described in (B). Cell lysates were analyzed by Western blotting employing antibodies against HER-2 protein or its phosphorylated form. Band quantitation of HER-2 protein was obtained densitometrically. Values were normalized to control values relative to cells incubated with DMSO. (D) Whole cell lysates were analyzed by Western blotting with antibodies directed against the indicated proteins. Densitometric analysis was performed on the indicated protein bands. All experiments were performed three times obtaining similar results and one representative experiment is shown. In all experiments, β-actin was detected as control for equal loading.

Next, it was of interest to examine whether the effects described above were associated with inhibition of EGFR and/or HER-2 activation as a number of studies suggest that aberrant expression of EGFR and HER-2 might be associated with multiple drug resistance in human pancreatic cancer cells (5,6). PANC-1 cells were cultured in serum-free medium for 24 h and then incubated with various concentrations of AS104 for 3 h before adding epidermal growth factor (100 ng/ml) for 10 min prior harvesting (Fig. 6A). Extracts were then analyzed by Western blotting using antibodies directed against phosphorylated EGFR. We found that cell treatment with AS104 did not affect the kinase activity of EGFR induced by incubation with EGF confirming observations reported earlier (16). The specificity of AS104 was also tested *in vitro* on a panel of 50 recombinant protein kinases including EGFR. As shown in Table I, AS104 did not exert any significant inhibitory effect on either EGFR or any other tested protein kinase except for CAMK1, FGFR3, VEGFR2 and PIM3 where the activity slightly decreased by 27.4, 39.1, 21.1 and 23.7%, respectively, with respect to control assays. However, experiments with cells incubated for 48 h with increasing concentrations of AS104 and stimulated with EGF before harvesting, showed a marked decrease in EGFR phosphorylation accompanied by a concomitant significant decrease in the total cellular levels of EGFR protein in cells incubated with 30 and 40 μM AS104, respectively (Fig. 6B). We extended the analysis to the expression and phosphorylation levels of HER-2 (Fig. 6C). As in the case of EGFR, incubation of cells with up to 40 μM AS104 markedly decreased the phosphorylation and protein expression levels of HER-2 with respect to DMSO-treated cells.

**Figure 7:**
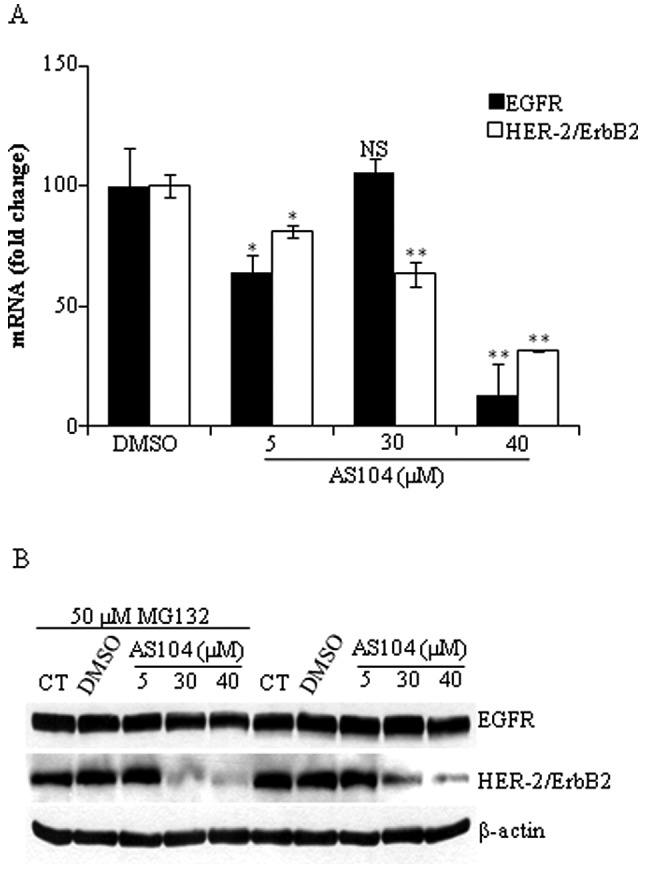
Treatment of cells with AS104 results in down-regulation of EGFR- and HER-2-mRNA expression levels. (A) PANC-1 cells were treated for 48 h with increasing concentrations of AS104. EGFR- and HER-2-mRNA was quantified (triplicate measurements) relative to β-actin by quantitative RT-PCR, respectively. Fold changes are relative to results from cells incubated with DMSO only. Error bars, SD; ^*^P<0.05; ^**^P<0.001; NS, not significant. (B) Cells were left untreated (CT), incubated with DMSO or various concentrations of AS104 for 24 h. Where indicated, cells were incubated with 50 μM MG132 for 6 h before harvesting. Whole cell lysates were analyzed by Western blotting with antibodies against the indicated proteins. Two separate experiments were performed obtaining similar results. Data from one representative assay are shown.

The mitogen-activated protein kinases (MAPKs) ERK1 and ERK2 belong to a protein kinase cascade downstream of the EGF receptor family-signaling and play a critical role in the regulation of cell proliferation, growth and survival (26–28). Another pathway associated with activation of members of the EGF-receptor family is the PI3K/AKT signaling cascade through which EGFR and HER-2 proteins provide survival signals to cells (28–30). The effects of EGFR- and HER-2 alteration were examined on basal MAPK and AKT activation in PANC-1 cells (Fig. 6D). Cells incubated with increasing amounts of compound showed reduced basal levels of phosphorylated MAPK to ~20% (p44MAPK) and ~31% (p42MAPK), respectively, at 40 μM AS104. MAPK protein levels did not vary with respect to untreated or DMSO-treated cells. The activity of AKT was analyzed under the same experimental conditions using phospho-specific AKT antibodies directed against phosphorylated Ser473 and Thr308 amino acids, respectively. Here, AS104 treatment led to reduction in the levels of phosphorylated AKT starting at 5 μM AS104. Interestingly, when we analyzed the phosphorylation status of Jun-amino-terminal kinase (JNK) whose role in cell death is well established (reviewed in ref. 31), we observed JNK activation/phosphorylation in cells incubated with 40 μM AS104 for 48 h suggesting that the JNK pathway might contribute to cell killing in pancreatic cancer cells treated with AS104. Next, to evaluate whether down-regulation of EGFR/HER-2 protein expression levels resulted from impaired transcriptional activity and/or accelerated degradation of the EGF receptor family members, EGFR- and HER-2-mRNA isolated from cells treated with increasing concentrations of AS104 for 48 h was subjected to quantitative RT-PCR analysis as described in Materials and methods. As shown in Fig. 7A, treatment of cells with AS104 markedly decreased EGFR and HER-2-mRNA levels in a dose-dependent manner. Cell incubation with 40 μM AS104 led to an 87% reduction in EGFR-mRNA level while in the case of HER-2 there was a 69% decrease in the mRNA level with respect to control experiment. In order to determine whether changes in the expression levels of both receptors were due to accelerated protein degradation, cells were incubated for 24 h with increasing concentrations of AS104 with or without a specific proteasome inhibitor (i.e., MG132) added to the incubation medium 6 h before harvesting the cells. As shown in Fig. 7B, the presence of MG132 did not result in the accumulation of EGFR and HER-2 suggesting that lower expression of EGFR/HER-2 was not due to accelerated protein degradation.

## Discussion

To date, pancreatic cancer is a malignancy with poor prognosis refractory to conventional therapy. Resistance towards chemotherapeutic treatment is due to alteration of multiple signaling pathways which are mitogenic, anti-apoptotic, pro-angiogenic and -invasion. Members of the ErbB family of transmembrane tyrosine kinase growth factor receptors, which includes EGFR and HER-2, are frequently overexpressed or constitutively activated in pancreatic adenocarcinoma and their expression has been shown to correlate with worse outcome and multiple drug resistance (32,33). In recent years, a number of strategies targeting these receptors have been developed with variable success (1,5). Multiple mechanisms that can account for the acquired or inherent resistance of pancreatic cancer have been reported supporting the notion that for a clinically relevant effect to be achieved, treatment strategies should consider targeting of multiple signaling pathways or multiple levels of a major pathway that sustain pancreatic cancer progression.

In the present study, we tested the cytotoxicity and growth inhibitory effects of a newly synthesized 2-triazenoazaindole compound, AS104, on a panel of four human pancreatic cancer cell lines. Incubation of cells with various concentrations of AS104 led to the finding that AS104 is able to induce significant cell death in a dose-dependent manner. We report evidence that AS104 leads to induction of both apoptosis and autophagy indicated by the fact that release of mitochondrial cytochrome c, cleavage of full length PARP and accumulation of LC3B-II proteins were observed under the applied experimental conditions. Autophagy and apoptosis may act independently, in parallel or influence one another (34). At present, the interplay between apoptosis and autophagy is elusive. Induction of autophagy may be necessary for apoptotic cell death. Alternatively, it may represent a protective mechanism activated in response to AS104 treatment. Although AS104 represents a novel triazene class of compounds with an azaindole moiety that was designed to inhibit EGFR tyrosine kinase activity, this compound failed to meet this initial expectation (16). Analysis of lysates from cells overexpressing EGFR with short-term treatment of AS104 revealed no changes in the phosphorylation levels of endogenous EGFR and hence its tyrosine kinase activity. Similar results were also obtained by measuring the activity of recombinant EGFR *in vitro*. However, unexpectedly, cell treatment with AS104 resulted in significant down-regulation of EGFR- and HER-2-mRNA levels (Fig. 7A). Transcriptional inhibition of EGFR and HER-2 receptor was accompanied by down-regulation of their protein expression levels, respectively, and decreased levels of activated MAPK and AKT indicated by reduction in their phosphorylation following treatment with AS104 (Fig. 6). Interestingly, phosphorylation of JNK was found up-regulated. JNK isoforms were initially described biochemically to be stress-induced protein kinases. Hence, increased phosphorylation of JNK observed following AS104 treatment might represent a stress response necessary for initiating the cell death signaling (31). Treatment of pancreatic cancer cells with AS104 leads to significant transcriptional down-regulation of *EGFR* and *HER-2* genes. Although treatment of cells with 30 μM AS104 results in enhanced EGFR-mRNA levels, an effect that remains to be addressed in future studies, it is likely that down-regulation of EGFR- and HER-2-mRNA levels might be accompanied by decreased expression of transcription factors controlling the synthesis of these receptors. This has been shown previously in bladder cancer cells where incubation with betulinic acid and curcumin down-regulated specificity protein (Sp) transcription factors and this was accompanied by decreased expression of EGFR mRNA and protein levels (35). Additionally, one cannot exclude the regulatory contribution of nuclear factor-κB (NF-κB). Activated NF-κB translocates to the nucleus resulting in the transcription of several genes, among which are cyclooxygenase COX-2 and EGFR (36,37). Inhibition of NF-κB activation by erlotinib and celecoxib combination treatment has been reported to result in significant apoptotic cell death and down-regulation of the transcription of EGFR and COX-2 enzyme (22).

Additional studies are required to determine the precise molecular mechanisms responsible for the anti-proliferative effects observed following cell treatment with AS104 as well as the mode by which AS104 induces decreased EGFR and HER-2 expression by blocking transcription of the corresponding genes. However, the observed biochemical selectivity of AS104 for both receptors together with its potent anti-proliferative effects makes this class of compounds attractive novel chemotherapeutic agents for the treatment of patients with pancreatic cancer.

## Figures and Tables

**Table I:** Specificity of AS104 [ethyl 2-(3,3-dibenzyl1-triazenyl)-1H-pyrido(2,3-c)pyrrolo-3-carboxylate].

Kinase	Activity (%)
ABL1	92.0
ABL2/ARG	96.6
AMPK (A1/B1/G1)	87.8
AMPK (A1/B1/G1)	92.5
ASK1/MAP3K5	106.6
Aurora A	100.1
BRAF	101.0
c-Kit	94.1
CAMK1a	72.6
CAMKK1	155.2
CAMKK2	101.7
CDK1/cyclin A2	93.5
CDK2/cyclin A	96.2
CHK1	104.4
CHK2	89.1
CK1e	106.6
CK2a	104.3
CK2a2	103.8
COT1/MAP3K8	92.5
DYRK1/DYRK1A	100.0
EGFR	103.0
EPHA1	94.5
EPHB4	87.9
ERBB4/HER4	97.5
ERK1	117.9
ERK2/MAPK1	115.4
FGFR1	86.8
FGFR2	97.7
FGFR3	60.9
FGFR4	97.9
FLT1/VEGFR1	100.0
FLT3	84.8
FLT4/VEGFR3	87.9
IGF1R	91.0
JAK3	85.6
JNK2	115.0
KDR/VEGFR2	78.9
LCK	88.1
LINK1	101.1
LKB1	178.6
LYN	94.7
MEK1	101.7
MKK6	94.7
MLK1/MAP3K9	95.4
MST1/STK4	90.8
mTOR/FRAP1	97.0
PIM1	86.8
PIM2	94.4
PIM3	76.3
RAF-1	101.6

Kinase assays were performed as described in Materials and methods.
